# Light-sheet Bayesian microscopy enables deep-cell super-resolution imaging of heterochromatin in live human embryonic stem cells

**DOI:** 10.1186/2192-2853-2-7

**Published:** 2013-11-20

**Authors:** Ying S Hu, Quan Zhu, Keri Elkins, Kevin Tse, Yu Li, James A J Fitzpatrick, Inder M Verma, Hu Cang

**Affiliations:** 1 Waitt Advanced Biophotonics Center, Salk Institute for Biological Studies, La Jolla, CA 92037, USA; 2 Laboratory of Genetics, Salk Institute for Biological Studies, La Jolla, CA 92037, USA; 3 Department of Bioengineering, University of California San Diego, La Jolla, CA 92093, USA

**Keywords:** Super-resolution imaging, Light sheet, Bayesian, Heterochromatin, Human embryonic stem cell

## Abstract

**Background:**

Heterochromatin in the nucleus of human embryonic cells plays an important role in the epigenetic regulation of gene expression. The architecture of heterochromatin and its dynamic organization remain elusive because of the lack of fast and high-resolution deep-cell imaging tools. We enable this task by advancing instrumental and algorithmic implementation of the localization-based super-resolution technique.

**Results:**

We present light-sheet Bayesian super-resolution microscopy (LSBM). We adapt light-sheet illumination for super-resolution imaging by using a novel prism-coupled condenser design to illuminate a thin slice of the nucleus with high signal-to-noise ratio. Coupled with a Bayesian algorithm that resolves overlapping fluorophores from high-density areas, we show, for the first time, nanoscopic features of the heterochromatin structure in both fixed and live human embryonic stem cells. The enhanced temporal resolution allows capturing the dynamic change of heterochromatin with a lateral resolution of 50–60 nm on a time scale of 2.3 s.

**Conclusion:**

Light-sheet Bayesian microscopy opens up broad new possibilities of probing nanometer-scale nuclear structures and real-time sub-cellular processes and other previously difficult-to-access intracellular regions of living cells at the single-molecule, and single cell level.

## Background

In the nucleus of eukaryotic cells, chromatin is organized into two distinct domains: the lightly packed and actively transcribed euchromatin, and the condensed and transcriptionally silent heterochromatin. Although transcriptionally repressive, heterochromatin is known to provide binding sites for regulatory proteins, and therefore, plays an important role in the epigenetic regulation of a variety of biological processes, such as development, cellular differentiation and maintenance of genome integrity (Maison & Almouzni 2004). Human embryonic stem cells (hESCs) are among the most commonly utilized pluripotent cells that hold promise in tissue replacement therapy for human diseases. Although hESCs are well characterized as being globally hyperactive in transcriptional activities, better characterization of the structure and dynamics of heterochromatin promotes in-depth understanding of their unique developmental potential which gives rise to different cell lineages, such as bone, muscle, and neural cells (Thomson et al. 1998). Recent developments in high-throughput DNA sequencing-based chromatin conformation capture assays suggest complex topographical organization of megabase-sized domain structures in the genome architecture (Dixon et al. 2012; Lieberman-Aiden et al. 2009). However, the sequencing techniques only provide averaged topological information from an ensemble of cells. Direct visualization of these domain structures in individual cells is a key step to advance the epigenetic study by bridging the genome architecture with the underlying regulatory processes. A viable imaging technique with high spatial and temporal resolution will fulfill this role.

Single-molecule super-resolution imaging is capable of extracting detailed structural information from biological samples in a physiologically viable environment and achieving a spatial resolution *on par* with the electron micrograph (Rust et al. 2006; Betzig et al. 2006; Hess et al. 2006). Key to achieving a high spatial resolution is a high signal-noise ratio (SNR) and a large number of identifiable single molecule events. On one hand, the localization width of a single-molecule event is inversely related to its SNR. On the other hand, because localizations are identified from a set of acquired images, traditional PALM and STORM techniques rely on thousands of individual imaging frames to achieve a lateral resolution of a few tens of nanometers. The acquisition typically takes several minutes, and has largely hindered this promising technique from gaining more popularity and providing mechanistic insights to biomedical studies.

The thickness of hESCs poses a major challenge to achieve high SNR with conventional imaging modalities (Patterson et al. 2010; Moerner 2007; Rust et al. 2006; Betzig et al. 2006; Hess et al. 2006). Individual hESCs have a large cell volume (Zwaka & Thomson 2003) and a nucleus that is thicker than 10 µm (Additional file 1: Figure S1). Total-internal-reflection microscopy (TIRF) is a widely adapted high-SNR technique for super-resolution imaging. However, TIRF has a limited probing depth of ~200 nm, covering only the basal membrane of a cell (Huang et al. 2008; Bates et al. 2007; Lippincott-Schwartz & Patterson 2009). Epi-illumination can reach deeper inside a cell at the cost of exciting unwanted molecules and poor SNR due to the generation of out-of-focus fluorescence. We resort to a selective-plane illumination strategy, which has been used for tissue-level imaging (Ahrens et al. 2013; Keller et al. 2010; Keller et al. 2008; Tomer et al. 2012) and single molecule tracking (Ritter et al. 2010; Friedrich et al. 2009), to improve the background for super-resolution imaging of thick samples.

Importantly, imaging live hESCs also requires a much faster imaging speed to counter cell motion and to capture dynamic changes that cannot be obtained by any other biotechnological means. Standard single-emitter fitting programs render the technique impractically slow (*i.e.* minute-scale acquisition times), even with the aid of the light-sheet illumination. Since the Nyquist sampling theorem requires approximately two data points per resolution unit to faithfully reconstruct an image (Shroff et al. 2008; Patterson et al. 2010), reducing the number of image frames requires increasing the number of resolvable single-molecule events per frame. Spatially overlapping molecules within a diffraction-limited focus volume in the same frame breaches the sparcity requirement of many single-emitter fitting programs (Henriques et al. 2010; Wolter et al. 2012) and further slow down the data acquisition. Here, we demonstrate a high-SNR illumination scheme that works hand in hand with a highly efficient reconstruction algorithm resolving overlapping fluorophores to enable rapid deep-cell super-resolution imaging.

## Methods

### Optical design for prism-coupled light-sheet illumination

**Figure 1:**
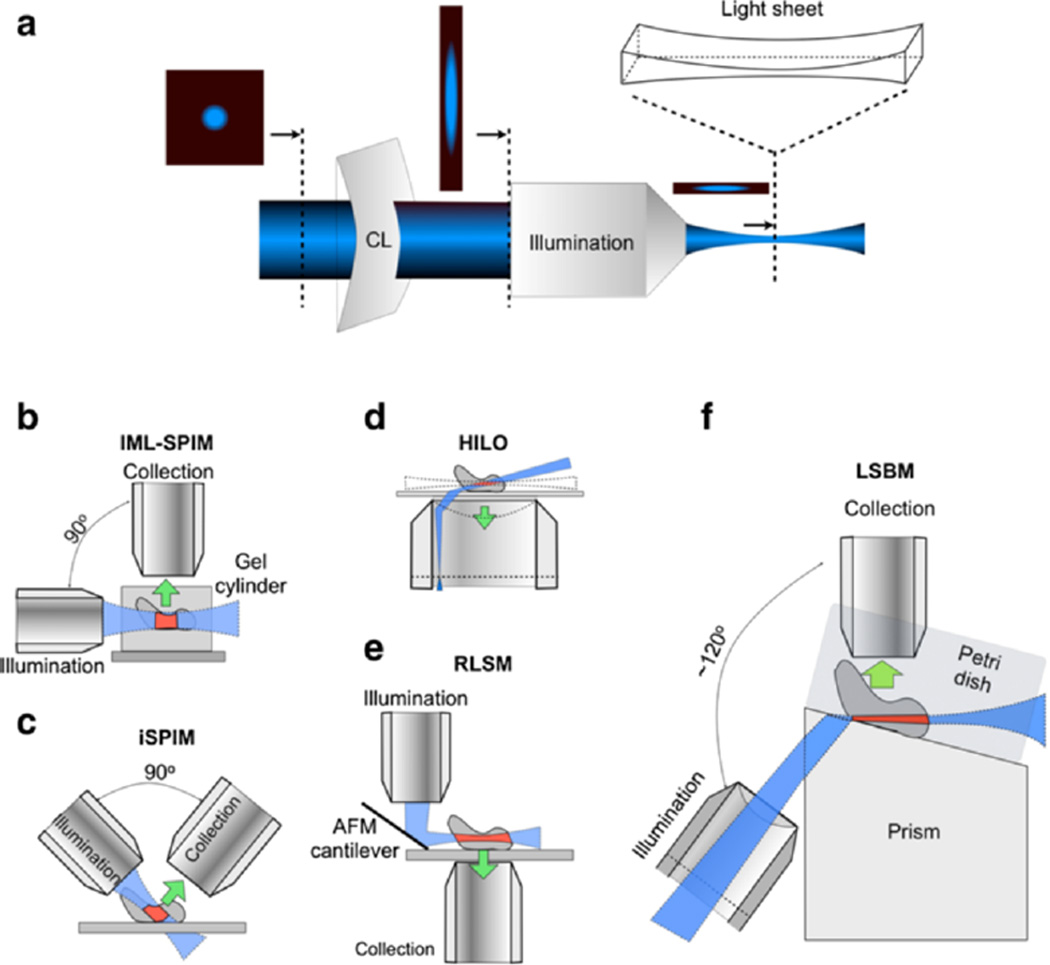
Illustration demonstrating (a) generation of the laser light sheet, and various light-sheet implementations including: (b) individual molecule localization-selective plane illumination microscopy (IML-SPIM), (c) inverted SPIM (iSPIM), (d) highly inclined laminated optical sheet (HILO), (e) reflected light-sheet microscopy (RLSM), and (f) prism-coupled light-sheet Bayesian microscopy (LSBM).

To reject out-of-focus fluorescence background from thick samples, selective-plane illumination microscopy (SPIM), also known as light-sheet microscopy, has been recently introduced for single-molecule super-resolution imaging by Zanacchi *et al* (Cella Zanacchi et al. 2011). The planar illumination is wide-field compatible and faster than scanning-based two-photon techniques for large areas (Folling et al. 2007; York et al. 2011; Vaziri et al. 2008). To generate a light sheet, laser light passes through a cylindrical lens and focuses to a line onto the back aperture of an illumination objective, which delivers the planar sheet of light to an imaging sample (Figure 1a). For imaging the intact nucleus with high speeds, light-sheet illumination provides optical sectioning with ease. While the concept of SPIM for single-molecule imaging has been successfully demonstrated, the original light-sheet condenser design has essentially remained unchanged since its conception by Siedentpf and Zsigmondy in 1903 (Siedentpf and Zsigmondy 1903). Considerable efforts have been devoted to searching for a method to induce light sheet illumination suitable for sectioning sub-cellular regions, while accommodating the use of a high-numerical-aperture (NA≥1.0) objective lens for high photon collection efficiency, and providing a large and flat field of view (FOV) to cover the entire region of interest. For example, the individual molecule localization SPIM (IML-SPIM) (Cella Zanacchi et al. 2011) and inverted SPIM (iSPIM) (Wu et al. 2011) (Figure 1b–c) impose a space constraint to simultaneously support a thin light sheet and a high-NA collection objective from the orthogonally oriented objectives. Alternatively, highly inclined and laminated optical sheet (HILO) uses a single high-NA objective; but its FOV is limited due to the oblique illumination (Tokunaga et al. 2008) (Figure 1d). A recent design by Gebhardt *et al.* attempted to address this problem by using a polished AFM cantilever as a mirror, which was placed close to the sample (Figure 1e). The mirror deflects the light sheet from the vertically-oriented illumination objective to generate a horizontally-oriented illumination plane coupled to a high-NA imaging objective with a slight horizontal offset on an inverted microscope (Gebhardt et al. 2013). This technique induces a 2-µm gap that cannot be imaged in the samples.

To adapt the light sheet illumination for imaging single hESCs, we have developed a simple and effective prism-coupled light-sheet condenser design. Demonstrated in Figure 1f and Additional file 2: Figure S2, light sheet was created by an *f* = 500 mm plano-convex cylindrical lens (Thorlabs LJ1144RM-A) and focused onto the back focal plane of an infinity-corrected Mitutoto M Plan APO 50× NA 0.55 condenser objective. A Pellin-Broca prism (Thorlabs ADBU-10) was placed after the condenser objective to redirect the light sheet horizontally onto a sample at the focal plane of an imaging objective, in our case, a Zeiss W Plan-Apochromat 63× NA 1.0 water immersion lens.

The prism-coupled condenser design enjoys three major advantages for super-resolution imaging. First, the de-coupling between the imaging and condenser objective and increase of the angle between them from 90 to roughly 120 degrees relaxes the space constraint for sample placement. The design widens the choice of imaging objectives and allows the use of even higher NA (NA > 1.0) water-immersion lenses. Second, the horizontal overlap between light-sheet illumination and the imaging plane yields a flat and large FOV with high SNR. Large FOV is necessary in order to capture the entire cross-section of the nucleus of the hESC. Third, compared to the AFM cantilever system (Gebhardt et al. 2013), the prism design is less prone to vibrations and simpler to implement in biology labs. In addition, the design is directly compatible with cell culture by accepting standard petri dishes. In our setup, we used a 35-mm glass-bottom petri dish (MatTek P35G-0-20-C) as the sample holder. For sample placement, we custom made an aluminum adaptor plate clamped onto a piezo translation stage (Thorlabs NanoMax-TS) and tilted at a horizontal angle of approximately 20 degrees (Additional file 1: Figure S1). The adaptor held the glass bottom dish flush with the top surface of the prism with index-matching oil in between. The piezo stage (Thorlabs NanoMax-TS) translated the sample along the top surface of the prism and exposed different depths of the hESC to light-sheet illumination. The excitation lasers, a 150-mW 561-nm diode-pumped solid-state laser (Cobolt Jive) and a 160-mW 642-nm diode laser (Coherent Cube), were brought together before the cylindrical lens and the condenser lens. Sharing the illumination path was a 60 mW 405 nm (Omicron PhoxX) laser for activation. For fluorescence imaging, an emission filter (600/60 for mEos and 705/130 for Alexa 647) and a notch filter were placed in front of an EMCCD camera (Andor iXon DU-897E with a pixel size of 16 × 16 µm). For imaging fixed cells, we used a custom mounting for the camera with a 300-mm tube lens, bringing the effective magnification to 115× (Additional file 2: Figure S2a). For live cell imaging, we adapted the light sheet system to the framework of a Zeiss Examiner.D1 upright microscope with mechanical support (not shown).

### Characterization of the light sheet

We used the RF module in COMSOL Multiphysics™ to perform the full-wave electromagnetic simulation of the light sheet. The incident Gaussian beam was characterized in the form:

$$E(r,z)=E_{0}\frac{w_{0}}{w(z)}\text{exp }[-\frac{r^{2}}{w{\left(z\right)}^{2}}]\;\text{exp }[-i(kz-{\text{tan}}^{-1}\frac{z}{z_{R}}+\frac{kr^{2}}{2R(z)})],$$

where *r* is the radial distance from the center of the beam, *z* is the axial distance, *w*_0_ is the beam waist and set to 2 µm, $w(z)=w_{0}\sqrt{1+{\left(\frac{z}{\pi w_{0}^{2}/\lambda }\right)}^{2}}$ is the radius at which the intensity of the beam drops to, 1/*e^2^*, $R(z)=z[1+{\left(\frac{\pi w_{0}^{2}}{\lambda z}\right)}^{2}]$ is the radius of the wave fronts. The Gaussian wave was launched from the bottom of the 2-D simulation domain with an excitation of the magnetic field in *z*. We plotted the intensity as the square of the normal *E* field. The FWHM from the line plot of the intensity profile characterized the width and length of the light sheet. For demonstration, we simulated the light sheet at 561 nm. The refractive index was 1.333 for water and 1.512 for glass.

For visualizing and measuring the thickness of the light sheet, we used fluorescent bead solution (Invitrogen F-8794) with 10^6^× dilution. A glass bottom dish containing 3 ml diluted fluorescent bead solution was use for profiling. The thickness was determined from the image captured by the EMCCD camera and the image pixel size.

### Sample preparation via transgenetic labeling or immunostaining

Human embryonic stem cells were cultured at the Stem Cell Core facility of the Salk Institute as previously described (Woods et al. 2011). Two NIH-registered hESC cell lines were cultured: WA01 (H1) and (Hues6). Both cell lines were determined to be karyotypically normal by cytogenetic analysis and shown to be pluripotent by in vivo teratoma histological assays. Pluripotent cell lines were cultured and expanded using Matrigel (BD Biosciences, San Diego, CA) to maintain their pluripotency. To generate transgenic cell lines, we constructed lentiviral vectors, pBOB-CAG-mEos2 and pBOB-CAG-mEos3, to express fusion proteins of mEos2-HP1α/mEos3-HP1α or mEos2-Centrin2/mEos3-Centrin2. Human embryonic stem cells were passaged by TrypLE digest, split into single cells, and subsequently cultured in the presence of Rock Inhibitor on Matrigel. Lentivirus infection was performed at a multiplicity of infection of 1:20 to 50 by spinning infection at a speed of 800 g for 1 h. Transduced cells were further incubated in fresh media for 48 h and seeded onto glass bottom dishes.

For immunostaining, we followed standard labeling procedures for STORM. Briefly, cells were fixed in 3% (v/v) paraformaldehyde (Alfa Aesar 43368) and 0.1% glutaraldehyde (Sigma G5882) (v/v) in PBS for 10 min and washed three times in filtered PBS. The remaining PFA and glutaraldehyde were reduced by incubating fixed cells in freshly prepared 0.1% (w/v) sodium borohydride (Sigma 213462) for 7 min, followed by permeabilization in the blocking buffer (5% (v/v) normal goat serum and 0.2% (v/v) Triton X100 (Sigma T8787) in PBS) for approximately one hour. The primary mouse anti-β-tubulin antibody (Invitrogen 1083461A) was diluted 200× in the blocking buffer with a 30-min incubation time. After washing five times in the washing buffer (0.2% (v/v) normal goat serum and 0.1% (v/v) Triton x100 in PBS), cells were incubated in the Alexa Fluor 647 conjugated goat anti-mouse IgG secondary antibody (Moleular Probes A21237) with 500× dilution in the blocking buffer for 30 min. The sample was protected from light and post-fixed with 3% (v/v) paraformaldehyde and 0.1% (v/v) glutaraldehyde in PBS for 10min after three-time wash.

### Imaging fixed and live cells

The image frame size was cropped to smaller than 128×128 pixels in order to increase the frame rate. Low excitation power was used to first locate the nucleus or microtubules, followed by imaging with full excitation power. The initial quick bleaching and subsequent blinking process were typically captured within the first 1–2000 frames with an exposure time of 30–50 ms. Standard imaging buffer was used for STORM: 40 mM D-glucose, 0.5 mg ml^−1^ glucose oxidase (Sigma G6125), 40 µg ml^−1^ catalase (Sigma C1345), and 143 mM β-mercaptoethanol (Sigma M6250) at pH 8.0. For vertical sectioning, the sample was translated tangential to the top surface of the prism by the piezo stage. With a known tilt angle, vertical translates can be precisely determined from the tangential movement.

The light-sheet microscope was adapted for short-duration live-cell imaging. Temperature was maintained by heating the imaging objective lens and glass prism using objective heaters (MTC-HLS-025/TC-HLS-025, Bioscience Tools, San Diego, CA). The imaging duration was less than 30 min. Transgenic Hues6 cells expressing mEos3-HP1α were seeded on glass bottom dishes with phenol-red free TeSR 30 min prior to imaging. For 250 ml of colorless TeSR, 500 µl of FGF-2, and 30 µl of TGFb1 were added to prevent cell differentiation. To minimize adverse phototoxicity, 1mW 405 nm laser was used to activate mEos3 for 5 s, followed by bleach-and-blink acquisition using the 561-nm laser at 40 mW for ~23 s with 4000 frames. Approximately 26 mW from the 561-nm laser and 200 µW from the 450-nm laser were delivered to the back aperture of the light sheet condenser objective. The exposure time was 50 ms and the frame size was 75×89 pixels. For imaging fixed cells, we used a custom-designed system and paired a 300- mm tube lens with a 63× Zeiss objective. The effective magnification was 114×, yielding a pixel size of 135 nm. For live cell imaging, we coupled light sheet into a commercial Ziess microscope with a 63× magnification, yielding a pixel size of 254 nm. A 2.5× magnifier can be used to further reduce the pixel size to 102 nm.

### Bayesian bleach-and-blink (3B) image reconstruction

The Bayesian method developed by Cox *et al.* (Cox et al. 2012; Rosten et al. 2013) globally analyzes sequential frames and applies a statistical model to infer the best possible distribution of molecules using their blinking and bleaching properties across different frames. Spatially overlapping fluorophores are allowed in any particular frames and molecules are resolved by *a priori* knowledge about the proceeding frames and *a posteriori* estimates of the bleaching and blinking of the molecules in the subsequent frames. Termed 3B for Bayesian bleaching and blinking analysis, the method harnesses single molecules from high-density areas and substantially shifts the burden from data acquisition to post-processing (Lidke 2012). Thorough discussion of the Bayesian theory and models of the bleaching and blinking properties of the molecules can be found in the literature (Cox et al. 2012; Rosten et al. 2013).

Prior to Bayesian analysis, we collected a set of standard STORM data using light-sheet illumination and analyzed the brightness and size distribution of the single-molecule events. The Bayesian code assumes a log-normal distribution, which closely characterizes our identified brightness (*R^2^* = 0.9982) and size (*R^2^* = 0.9957) values of the single-molecule events (Additional file 3: Figure S3). In comparison to the exponential distribution commonly used for single-molecule analysis, the observed log-normal distribution is attributed by the thresholding in localization analysis and the fact that brightness and lifetime measurements are frame-rate limited and typically do not cover the entire lifespan of the molecule.

We ported the Bayesian bleach-and-blink code to a supercomputing cluster Hopper at the National Energy Research Scientific Computing (NERSC) Center at the Lawrence Berkeley National Laboratory. For image reconstruction, over-exposed images at the beginning of the acquisition were discarded and subsequent 400 images (saved as fits files) were selected for processing. The 3B analysis was parallelized by dividing the image into 10-by-10-pixel sub-masks, each with a 50% horizontal and vertical overlap with its neighbors to eliminate edge artifacts. A Python-MPI wrapper was used to distribute the Bayesian calculation to a number of processors equal to the number of sub- masks. For a 128-by-128-pixel image, approximately 2,500 CPU hours were consumed. Details of the Bayesian algorithm and its implementation are discussed in Additional file 4: Note S1. We also provided an alternative computing resource openly distributed *via* Amazon and using its Elastic Cloud Computing (EC2) service for labs without the access to supercomputing resources (Hu et al. 2013).

## Results and discussion

### SNR enhancement

**Figure 2:**
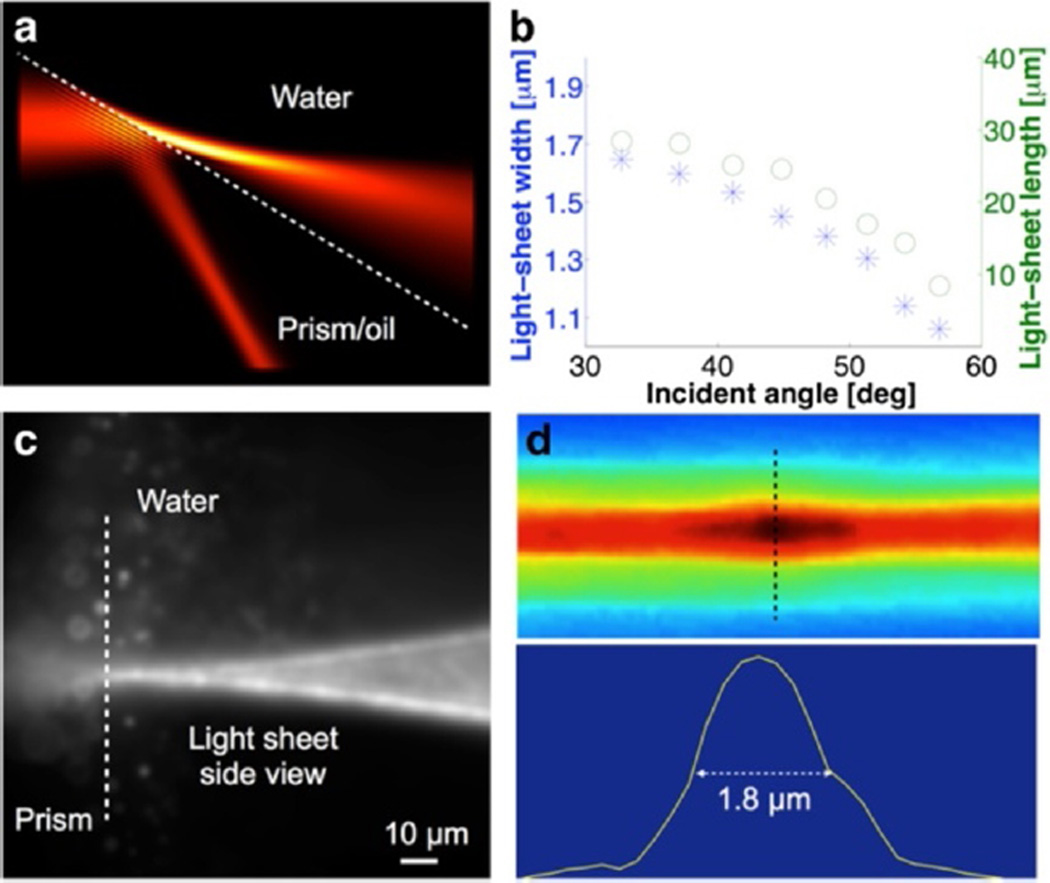
Characterization of the light sheet profile **(a)** Electromagnetic simulation of the intensity of a focused Gaussian beam at the interface. **(b)** Width and length of the light sheet at various incident angles at the interface calculated from the intensity profile obtained from electromagnetic simulations. Light-sheet width was calculated from the FWHM of the intensity profile, light-sheet length was calculated from the glass-water interface along the light sheet to the point where its intensity drops by one half. Glass prism with a refractive index of 1.515 and water with a refractive index of 1.333 were used for the simulation at 561 nm. **(c)** Side view of the light sheet focused at the interface where it enters water. **(d)** Side profile of the light sheet focused in water, *i.e.* further away from the interface, and the measurement of its thickness from the cross line profile.

We used finite-element simulations to help tailor the design parameters for imaging the hESC nucleus, which is ~10-µm thick and wide. As a first-order approximation, the thickness of the light sheet is determined by the diffraction-limited waist size of a focused Gaussian beam, *w*_0_, and its length *b* determined by $b=2\pi w_{0}^{2}/\lambda$. Reducing the thickness by one half shortens the length of light sheet and the effective FOV by four. We aimed to reduce the thickness of the light sheet to less than 1/5 of the thickness of the nucleus while maintaining an effective range covering the entire cross-section of the nucleus. We simulated the light sheet at a 20 degree top-surface slope of the prism (Additional file 5: Figure S4) and found the thickness to be ~1.1 µm and length to be ~14 µm (Figure 2a,b). By turning the elliptical beam 90 degrees (Figure 2c), we measured the FWHM of the light sheet to be ~1.8 µm (Figure 2d), which is in general agreement with the calculation. The slightly wider measured profile is likely due to 3^rd^ order aberrations, such as coma, induced at the interface, as well as the refractive index of the imaging medium (Additional file 6: Figure S5, Additional file 7: Note S2). In addition, the finite PSF of the microscope broadens the measurements on the thickness of the light sheet at its thinnest portion. A PSF of 0.25 µm of the microscope, for instance, increases the measured thickness by 0.25 µm. Thus, the actual thickness of the light sheet would be ~ 1.55 µm.

**Figure 3:**
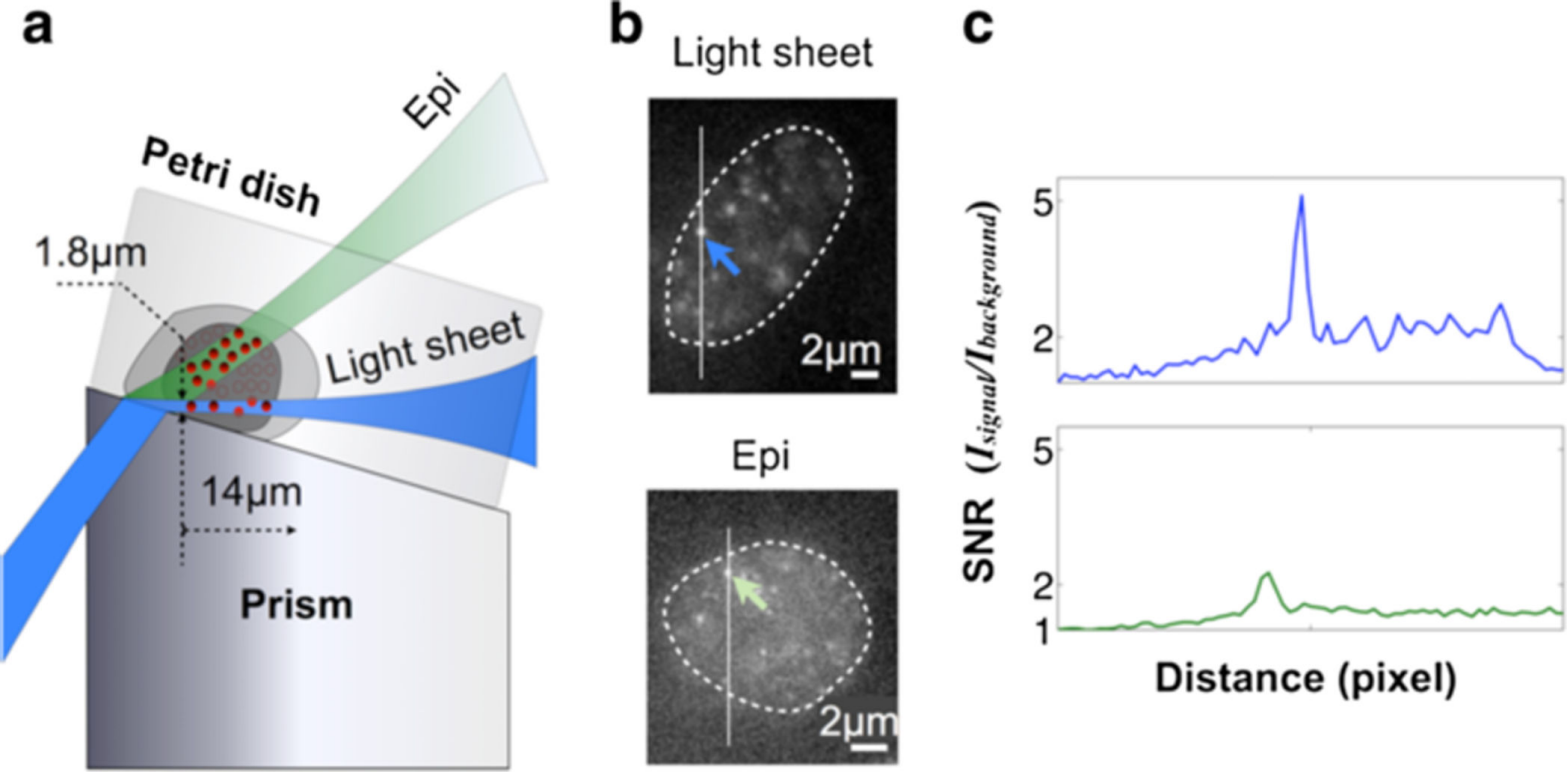
Light sheet illumination provides enhanced SNR for single-molecule imaging in the nucleus **(a)** Schematic depiction of the light-sheet and epi-illumination. **(b)** Single-molecule images collected from light-sheet (top) and epi-illumination (bottom) of the nucleus of fixed Hues6 (hESC) cells expressing mEos3 on HP1α. **(c)** Cross profiles demonstrating the enhanced signal-to-noise ration of the single-molecule event from light-sheet (top) *vs.* epi-illumination (bottom).

The enhanced SNR using light sheet illumination can be observed from single-molecule images. As a comparison, we performed single-molecule imaging of fixed mEos3-HP1α-labeled transgenic Hues6 hESCs using high-angle epi and prism-coupled light sheet illumination (Figure 3a). Figure 3b shows the raw single molecule imaging data averaged over 10 frames for data collected from the light sheet and epi using the same laser power and integration time. The SNR, defined as the ratio between the intensity of a single molecule event and of the background, is ~2 with epi, and ~5 with the light sheet illumination (Figure 3c). This high SNR improves the single-emitter fitting precision, and enhances the efficiency of the reconstruction algorithm for resolving more events per frame.

### Super-resolution imaging using LSBM

The Bayesian algorithm effectively resolves sub-diffraction cellular structures. We cross-compared the reconstructed structures between Bayesian and standard single-emitter fitting analysis using standard epi and light-sheet illumination for both thin and thick cell samples. Additional file 8: Figure S6 illustrates super-resolution heterochromatin foci structure in a fixed BJ fibroblast cell with epiillumination. Both the Bayesian and PALM approach revealed sub-diffraction clusters in each focus. Additional file 9: Figure S7 shows microtubule structures in a fixed H1 cell with light-sheet illumination. Because of the high background generated by out-of-focus excitation, the epiillumination with PALM analysis yielded relatively low resolution, while the Bayesian analysis with light-sheet illumination resolved much thinner fiber structures with higher resolution. We note that Bayesian analysis yields heterogeneous resolution, which is exaggerated in long and linear structures, such as microtubule fibers. Since the number of molecules in each frame is an estimated and non-negative variable in the Bayesian algorithm, its uncertainty leads to uneven thickness of the reconstructed fiber image. Nonetheless, the Bayesian method drastically outperforms other reconstruction programs when the same number of frames is used.

**Figure 4:**
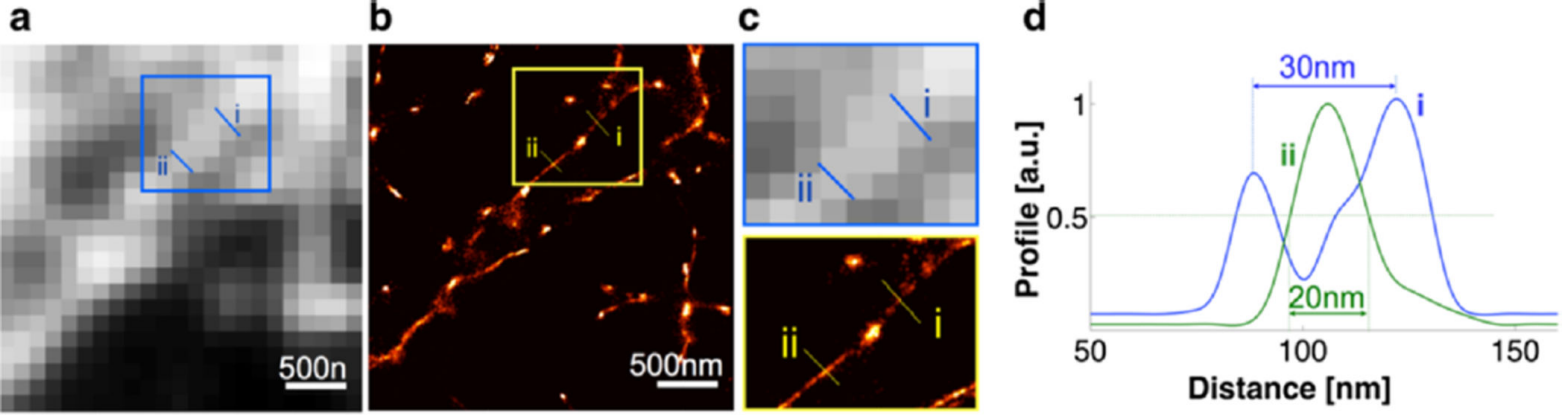
Bayesian reconstruction of microtubule fibers **(a)** Conventional fluorescent image of the microtubules in a fixed U2OS cell. **(b)** Bayesian reconstruction of the superresolution image of **(a)** obtained from 642-nm light sheet illumination. **(c)** High-magnification view of the highlighted regions in **(a)** (upper panel) and **(b)** (lower panel). **(d)** Cross profile for fiber sections outlined in the lower panel in **(c)**. The light sheet Bayesian microscope resolves 20-nm fibers separated by a distance of 30 nm, demonstrating its lateral resolution of 20–30 nm with fixed samples.

Albeit substantial reduced number of image frames, the Bayesian analysis can achieve comparable resolution to standard PALM/STORM techniques. Figure 4a,b show a conventional fluorescence image of microtubules in a fixed U2OS cell and a super-resolution image obtained from LSBM using 400 frames of the bleach-and-blink data. The effective resolution is evaluated as the spacing between adjacent fibers that are not resolvable in the conventional image (i) and the cross-section of the tubulin fiber (ii) (Figure 4c). We show in Figure 4d that LSBM resolves 20-nm microtubules and 30-nm spacing between the fibers at the diverging fork. This 30-nm lateral resolution is consistent with standard PALM/STORM techniques reported in the literature (Huang et al. 2008). We further performed super-resolution imaging of tubulin fibers in thick H1 cells (an hESC cell line) and engaged mother-daughter centrioles in transgenic retinal pigmentation epithelial (RPE) cells expressing photoactivatable protein mEos2 with a similar lateral resolution. These structures are located far deep into the cell for TIRF but can be readily reached by light sheet illumination. We show in Additional file 10: Figure S8 that these known structures are still resolvable by LSBM. More importantly, the Bayesian method drastically reduces the number of frames from tens of thousands to hundreds, significantly increasing the temporal resolution for live cell imaging.

### LSBM imaging of heterochromatin in fixed and live hESCs

**Figure 5:**
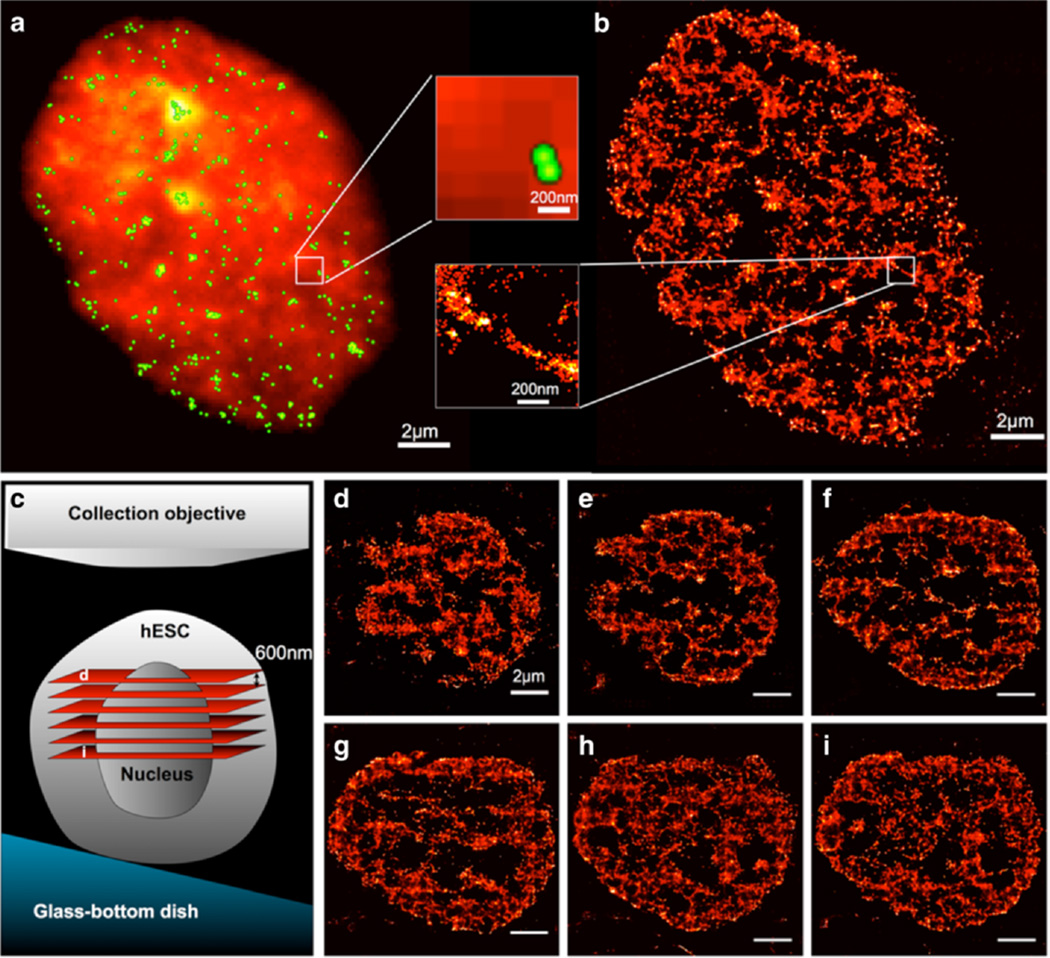
Light sheet Bayesian microscopy reveals the distribution of heterochromatin protein HP1α in an H1 cell **(a)** Frame-averaged conventional fluorescent image of the distribution of mEos-tagged heterochromatic protein HP1α in a hESC (H1). Green dots are localizations generated by *quickPALM* using the same imaging data. **(b)** Bayesian reconstructed super-resolution image of the same region demonstrating distinct regions of HP1α. Inset panels contrast marked difference in resolution. **(c)** Schematic illustration of the light sheet sectioning at various depths of the nucleus. **(d)–(i)** Sections of super-resolution images of HP1α in the nucleus of an H1 cell. Conserved topographical structures were observed at different planes 600-nm apart in the axial direction.

We generated transgenic hESC cell lines expressing photoactivatable proteins mEos2 or mEos3 on the heterochromatin protein HP1α using lenti-viral infection. Known to be enriched at the heterochromatic sites and maintaining the stability of heterochromatin, HP1α is regarded as a hallmark of heterochromatin (Cheutin et al. 2003). We performed imaging of the HP1α distribution in two different hESC cell lines: H1 (for fixed-cell imaging) and Hues6 (for live-cell imaging). Figure 5a shows a representative frame-averaged conventional fluorescent image of HP1α in a fixed hESC. One can observe a non-uniform distribution of HP1α throughout the nucleus. However, the overall structure appears diffusive and featureless (Figure 5a without green dots). The lack of distinct heterochromatin domains appears to be consistent with the fact that hESCs have globally hyperactive transcriptional activities. Comparatively, the super-resolution image generated from LSBM reveals distinct nanometer-scale features of heterochromatin in the entire cross-section of the nucleus (Figure 5b). The insets of Figure 5 illustrate a striking nanoscopic structure resolved by LSBM but not discernible in the conventional fluorescent image. We emphasize that the 3B algorithm is crucial for achieving the reported lateral resolution with only hundreds of frames. As a comparison, analysis using conventional single-molecule imaging methods, such as quickPALM (Henriques et al. 2010) or rapidSTORM (Wolter et al. 2012), with the same bleach-and-blink image frames, would only resolve sparse single-molecule events and fail to reconstruct the nanoscopic heterochromatin features (green dots in Figure 5a). The sparse localization is resulted from the program discarding most of the overlapping fluorophores.

The pattern of HP1α distribution was consistently found at various depths of the nucleus (Figure 5c–i). By utilizing the slope of the sample placement, we achieved vertical sectioning by horizontally translating the sample *via* a piezoelectric stage (Online methods). As the light-sheet illumination moves from the top towards the bottom of the nucleus, the cross-section of the nucleus gradually increases. In addition to the mesh-like structures, one can observe void areas of heterochromatin near the center of the nucleus. These morphological features suggest organized heterochromatin compartments within the nucleus of the hESC.

**Figure 6:**
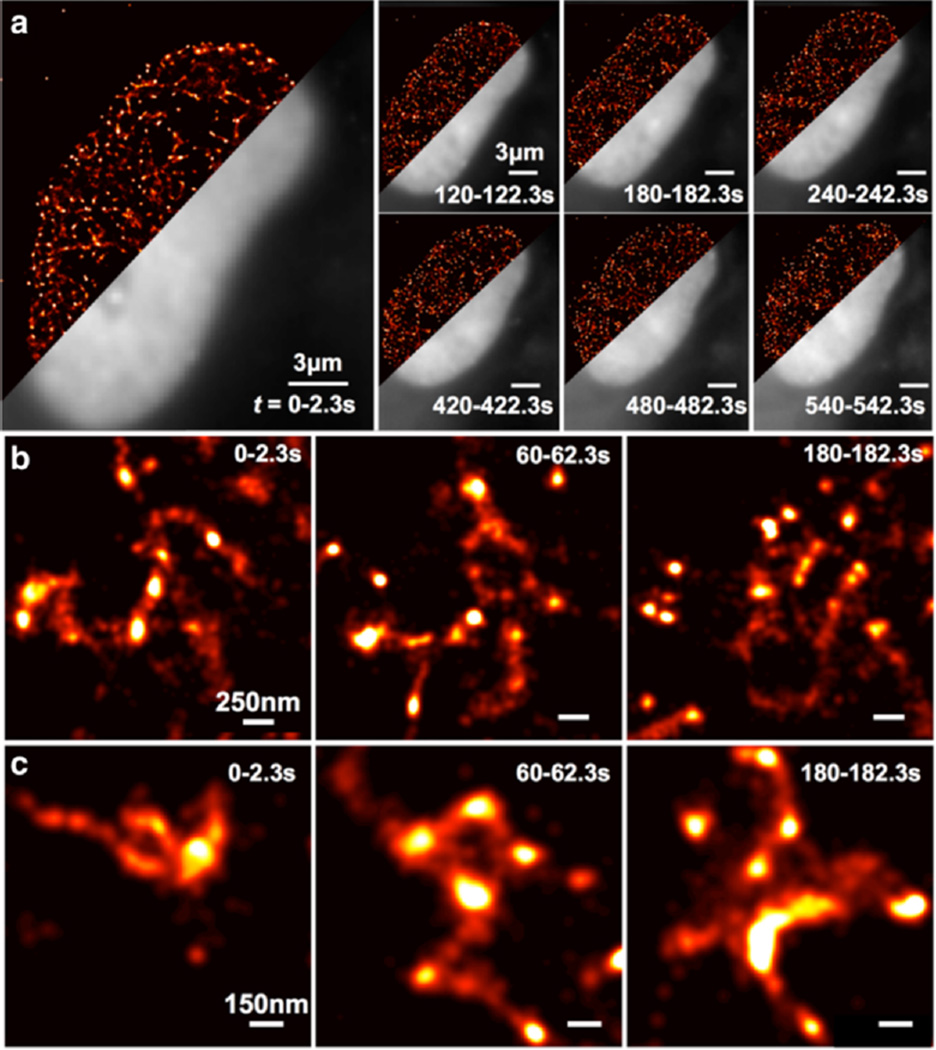
Super-resolution live cell imaging of the dynamics of HP1α in a Hues6 cell **(a)** Light-sheet imaging of HP1α in a living Hues6 cell with 2.3-s data acquisitions. Grey areas denote the frame-average conventional fluorescent images and the red areas denote the superresolution images of the HP1α structure. Acquisition time points are as marked. **(b)–(c)**, High-magnification views of the temporal dynamics of the HP1α distribution at two positions within the nucleus.

With the enhanced temporal resolution, we demonstrate live-cell imaging of heterochromatin in hESCs using LSBM. We adapted the LSBM for live-cell imaging by maintaining the temperature of the glass prism and the imaging objective (Online methods). We continuously delivered activation power followed by bleach-and-blink measurements, and reconstructed super-resolution images using 400 cropped frames captured within 2.3s every minute for approximately 15 minutes (Online Methods). Time-lapsed conventional fluorescent images showed noticeable change to the morphology of the nucleus on the minute-scale (grey areas in Figure 6a). For instance, the length of the cross section changed from 21 µm at 2 s to 18.5 um at 542 s. LSBM quickly resolved nanometer-scale heterochromatin structures within 2.3 s, as demonstrated by red areas in Figure 6a as well as with greater details in Figure 6b,c. The lateral resolution allowed resolving 50–60 nm features and dynamic differences between time points. Fast data acquisition mitigates the adverse effect from instrument drift and cell motion. In the near future, we expect to couple the Bayesian algorithm with a fast sCMOS camera and achieve super-resolution imaging over 10 fps. (Huang et al. 2013).

## Conclusions

Acquiring sub-cellular super-resolution images deep inside a living cell is one of the most challenging tasks in bioimaging. In this study, we designed LSBM and demonstrated its capability by visualizing the structure and dynamics of heterochromatin in fixed and live hESCs. With the fixed hESCs, we first demonstrated a mesh like heterochromatin structure in pluripotent stem cells. These structurally distinct organizations are also aligned with the topological domain hypothesis (Lieberman-Aiden et al. 2009; Dixon et al. 2012). The latest sequencing data suggest that mammalian genomes are organized into conserved topological chromatin-chromatin interaction domains for efficient large-scale gene expression regulation and constraining the spread of heterochromatin in different cell types (Dixon et al. 2012). The dynamic change of the nanoscopic structural features revealed in Figure 4 is a combination of the Brownian motion of the chromatin, the movement of heterochromatin in response to sub-cellular activities, and the cell motion (Dion & Gasser 2013). LSBM provides a viable imaging tool to examine these dynamics, for the first time, with nanometer resolution. Further studies can be done to examine the heterochromatin structure and its dynamics in hESCs at different phases of the cell cycle and during their responses to different differentiation stimuli. Techniques can be applied to label a region of interest to help lock the imaging FOV in order to counter cell motion. The 2.3-s temporal resolution of LSBM is a unique capability enabled by the Bayesian algorithm. Importantly, using bright and fast-switching molecules, and increasing the frame rate of the camera, *i.e.* by using a cropped mode for a smaller FOV, can further increase the imaging speed to the sub-second level.

Our prism-coupled light sheet design provides high SNR for imaging thick hESCs and accommodates a high-NA imaging objective, which is crucial for the photon collection efficiency for single-molecule super-resolution imaging. The majority of light sheet designs use an imaging lens with an NA of 0.8 or lower. However, a mere increase of 0.2 in NA from 0.8 to 1.0 increases the light collection efficiency by 32%. Although we demonstrated single-molecule super-resolution imaging through 10-µm thick nuclei in hESCs, the use of LSBM can be readily expanded for general biological samples more than a few micrometers thick, such as thin tissue slices, without modification. In fact, the robustness and ease of implementation is a significant advantage over other light-sheet techniques. Taken together, LSMB enables a wide array of *in vivo* studies from deep-cell imaging of sub-cellular structures to real-time probing of dynamic cellular processes with nanometer resolution.

## Footnotes

**Additional files** **Additional file 1: Figure S1**. Laser scanning confocal microscope (LSM) sectioning images comparing the thickness of the nucleus between a somatic cell (BJ fibroblast) and a human embryonic stem cell (Hues6). The transgenic cells express HP1α-mEos3. While the nucleus of the BJ fibroblast is ~ 3µm thick, the nucleus of an hESC is 3× thicker approaching 10 µm. **Additional file 2: Figure S2**. a) Pictorial depiction of the light sheet Bayesian microscope. Top left panel: a photograph demonstrating the prism-coupled light sheet set-up; right panel: SolidWorks™ rendering showing details of the prism placement with respect to the objectives. b) Schematic diagram of the optical setup. DM: dichroic mirror; BE: beam expander; CL: cylindrical lens; M: mirror; P: glass prism; GBD: glass bottom dish; TL: tube lens; F: emission and notch filters. **Additional file 3: Figure S3**. Measured log-normal distribution of the brightness and size of single-molecule events with light-sheet illumination. **Additional file 4: Note S1**. Implementation of the Bayesian bleach-and-blink (3B) algorithm. Typically, a sample of interest is quickly bleached out using high laser intensity and the first 3–400 image frames are used for reconstruction. There are highly overlapping fluorophores at the beginning of the acquisition. After the initial quick bleaching, only a small subset of the molecules remains on in a stochastic fashion. Fitting the bleaching and blinking pattern allows extraction of considerably more single-molecule events from high-density areas. Thus, it achieves a comparable spatial resolution with fewer image frames. We parallelized the Bayesian calculation by dividing the 400-frame tiff file into smaller 10-by-10-pixel sub-areas. Each adjacent sub-areas had a 5-pixel overlap in the horizontal and vertical direction. The spatial overlap reduces the edge artifact of the code when the boundary crosses through a high-density area. The parallelization allowed us to reconstruct larger image areas using cluster computing and super-computing resources. A minimum of 200 iterations were performed before the Bayesian algorithm was terminated. **Additional file 5: Figure S4**. Electromagnetic simulation of the intensity of the light sheet at the prism/oil – water interface. Figures (a) – (d) demonstrate the light sheet from being total-internal-reflected to being refracted into water. Incident and reflected Gaussian beams interfere and form interference pattern near the interface. This interference pattern can be clearly visible when imaging light sheet using fluorescent bead solution. The pattern also helps locating the interface when tuning the profile of the light sheet. The excitation wavelength is 561 nm. **Additional file 6: Figure S5**. Finite element simulation results showing the effect from index change of the medium on the light sheet. At the same incident angle of 27.5 degrees, and with the same incident light. (a)–(c) Finite element simulation showing the intensity of a focused Gaussian beam traveling from glass to a medium with a refractive index of 1.40, 1.35, and 1.30, respectively. (d) Length and width of the light sheet as a function of the medium index. Light-sheet width was calculated from the FWHM of the intensity profile, light-sheet length was calculated from the glass-water interface along the light sheet to the point where its intensity drops by one half. **Additional file 7: Note S2**. Light sheet characterization with different imaging media (refractive indices). For imaging fixed cells in PBS, which has a refractive index of 1.337 at 510 nm (Templer and Leatherbarrow 2002), close to the water index of 1.333, we do not expect significant change of the light sheet profile. For higher index changes, we characterized the effect of refractive index using a finite-element simulation. Additional file 6: Figures S5a–c plot the light sheet profile at three different indices. We expect the light sheet to bend up in high-index media, as described by the Snell’s law. This will reduce the overlap between the light sheet and the imaging plane and thus decrease the FOV. In addition, index change affects the width and length of the light sheet. We measured width as the FWHM at the beam waist and length as the distance from the interface where light enters the medium to the point along its propagation where the intensity drops by a half. As the index decreases, the light sheet will tilt towards the interface and its length generally decreases. Its width, however, first increases (*i.e.*, n>1.38) because a lower refractive index confined light in a lesser degree, and then decreases (*i.e.*, n<1.38) because of the NA limited by the interface, *i.e.*, intensity distribution is confined by the interface. In this particular simulation, broadening of ~100 nm and elongation of ~7 µm are expected when the medium index changes from 1.33 to 1.40. These changes increase the FOV and counter the reverse effect from the refractive angle. As indicated by the simulation, for live cell experiments, the incident angle has to compensate for the higher index from the cell medium. **Additional file 8: Figure S6**. Validation of the 3B code with PALM by imaging heterochromatin foci in a transgenic BJ fibroblast cell expressing mEos2 on HP1α. (a) Frame-averaged conventional image of the heterochromatin foci in a fixed BJ fibroblast cell. (b) High-magnification diffraction-limited view of the two foci. (c) Super-resolution images of the foci in (b) using a standard single-emitter fitting program. (d) Super-resolution images of the foci in (b) using the Bayesian method. (e) Line profiles showing sub-diffraction features resolved by the two reconstruction methods. **Additional file 9: Figure S7**. Super-resolution images of microtubule fibers with light sheet illumination reconstructed from (a) single molecule localizations using single-emitter fitting; (b) Bayesian analysis. **Additional file 10: Figure S8**. Comparison of conventional fluorescence images and Bayesian superresolution images. (a–b) Conventional and Bayesian-reconstructed super-resolution images of the microtubule fibers in H1 hESCs. (c) conventional fluorescent image of two adjacent centrioles in retinal pigmentation epithelium (RPE) cells (left) and Bayesian-reconstructed super-resolution image of the same region (right). Centrioles are tagged with mEos2 protein *via* trangenesis. While in the diffraction limited image, each centriole structure appears to be composed of two sub-units, the super-resolution image clearly demonstrates the mother and daughter centrioles that are still engaged.

## Abbreviations

LSBM: Light-Sheet Bayesian Microscopy
3B analysis: Bayesian bleach-and-blink analysis
hESC: Human embryonic stem cell
SNR: Single-to-noise ratio
TIRF: Total internal reflection fluorescence microscopy
FOV: Field of view
SPIM: Selective-plane illumination microscopy
IML-SPIM: Individual molecule localization selective-plane illumination microscopy
iSPIM: Inverted selective-plane illumination microscopy
HILO: Highly inclined and laminated optical sheet
AFM: Atomic force microscopy
EMCCD: Electron multiplying charge coupled device
RF: Radio frequency
FWHM: Full width at half maximum
NA: Numerical aperture
STORM: Stochastic optical reconstruction microscopy
PALM: Photoactivated localization microscopy
Amazon EC2: Amazon elastic compute cloud
